# The clinical profiles and outcomes of HIV-negative cryptococcal meningitis patients in type II diabetes mellitus

**DOI:** 10.1186/s12879-021-05867-5

**Published:** 2021-02-27

**Authors:** Hang Li, Xiaojing Li, Lei Zhang, Wenjie Fang, Keming Zhang, Amir Arastehfar, Macit Ilkit, Dongying Hu, Xianzhen Chen, Huiwei Wang, Liyan Ling, Jianjun Lin, Bin Xu, Wanqing Liao, Weihua Pan, Qilong Zhang

**Affiliations:** 1 Shanghai Key Laboratory of Molecular Medical Mycology, Shanghai Changzheng Hospital, Second Military Medical University, Shanghai, 200003 P. R. China; 2 Department of Dermatology, Shanghai Changzheng Hospital, Second Military Medical University, Shanghai, 200003 P. R. China; 3 Department of Dermatology, Affiliated Hospital of Hebei University of Engineering, Handan, Hebei P. R. China; 4 Westerdijk Fungal Biodiversity Institute, Utrecht, The Netherlands; 5 Faculty of Medicine, Çukurova University, Adana, Turkey; 6 Department of Laboratory Medicine, Pinghu Second People’s Hospital, Zhejiang, P. R. China; 7 Department of Clinical Laboratory, The Affiliated Xiangshan Hospital of Wenzhou Medical University, Ningbo, Zhejiang P. R. China; 8 Department of Neurology, Jiangxi Chest Hospital, Jiangxi, 330000 P. R. China; 9 Department of Neurology, Jiangxi Provincial people’s Hospital, Nanchang, 330006 P. R. China

**Keywords:** Cryptococcal meningitis, Type II diabetes mellitus, Clinical profile, Prognosis

## Abstract

**Background:**

The clinical profiles and outcomes of cryptococcal meningitis have been shown to vary depending on the underlying condition. The aim of this study was to investigate clinical characteristics and outcomes in patients with and without type II diabetes mellitus.

**Methods:**

A retrospective study was performed. Clinical data of HIV-negative cryptococcal meningitis patients with type II diabetes mellitus (*n* = 26) and without type II diabetes mellitus (*n* = 52) referring to the Jiangxi Chest Hospital between January 2012 to December 2018 were analyzed. The data were analyzed using chi square, none-parametric tests, and logistic regression. *P*-values < 0.05 were considered significant.

**Results:**

In this study, cryptococcal meningitis patients suffering from type II diabetes mellitus had a higher mortality (23.08% vs. 7.69%; *P* = 0.055), and required longer hospitalization (59.58 vs. 42.88 days; *P* = 0.132). Moreover, cerebrospinal fluid examinations revealed that cryptococcal meningitis patients with type II diabetes mellitus had higher opening pressure (271.54 vs. 234.23 mmH_2_O; *P* = 0.125).The results of multivariate regression analysis revealed that cryptococcal meningitis patients with type II diabetes were more often presented with visual disorders (28.54% vs. 11.54%; [95% CI 0.056–0.705]; *p* = 0.012), and had higher cerebrospinal fluid protein levels (1027.62 ± 594.16 vs. 705.72 ± 373.88 mg/l; [95% CI 1.000–1.002]; *p* = 0.016). Among patients with type II diabetes mellitus, nausea and vomiting was more frequent at the initial visit in those died (100% vs. 50%; *p* = 0.027), and 66% of died type II diabetes mellitus patients were poorly controlled blood glucose level, compared with 30% in survival type II diabetes mellitus patients.

**Conclusion:**

This study suggests that cryptococcal meningitis patients with type II diabetes mellitus differ significantly from cryptococcal meningitis patients without type II diabetes mellitus with respect to clinical symptoms such as visual disorders and cerebrospinal fluid examination. The presence of nausea and vomiting among type II diabetes mellitus patients could have implication in mortality.

## Background

Cryptococcal meningitis (CM) is a serious central nervous system infection caused by *Cryptococcus neoformans* and *Cryptococcus gattii* complexes [1], and cryptococcal meningitis is a leading cause of death among HIV-infected individuals, especially in Sub-Saharan Africa [2, 3]. Risk factors for cryptococcal infection include: HIV infection, transplant recipients, malignancy, and corticosteroid use. Due to the introduction of effective antifungal drugs and improved management of HIV, the incidence and mortality of cryptococcal meningitis in HIV-infected patients has decreased in recent years. However, the increasing population of HIV-negative patients with immunocompromised conditions has received increasing attention [4–7]. Importantly, approximately 80% of cryptococcal meningitis cases in China and the US were reported in HIV-negative populations [8, 9]. Many of these patients have underlying diseases, such as cancer, systemic lupus erythematosus, type II diabetes mellitus (DM II), nephrotic syndrome and tuberculosis [4–7]. Of note, the impact of various clinical features on survival of cryptococcal meningitis in HIV-infected individuals have been adequately described in previous studies [10, 11]. Moreover, some studies have investigated the clinical presentation and therapeutic outcomes in HIV-uninfected populations [6, 12], but comparative assessments of cryptococcal meningitis patients with and without underlying diseases are limited.

Type II diabetes mellitus is a group of metabolic disorders characterized by a long-term elevation of blood glucose levels. China has the world’s largest type II diabetes mellitus population; according to the latest national survey, 11.6 and 50.1% of Chinese adults have diabetes and prediabetes disorders, respectively, accounting for nearly 25% of the global diabetes population [13, 14]. Inadequate glucose management in diabetic patients increases their risk of infections and usually requires more hospitalization compared with that of the general population [14–16]. The prevalence of diabetes mellitus in cryptococcosis patients varied from 2 to 25%, based on previous studies, and the diagnosis of diabetes mellitus was also associated with 1-year and overall mortality in cryptococcosis populations [17–19]; therefore, understanding the differences of clinical manifestations between cryptococcal meningitis patients with and without type II diabetes mellitus may have clinical implications and contribute in ameliorating survival rate. Consequently, we conducted a retrospective study and evaluated the clinical profiles of HIV-negative cryptococcal meningitis patients with and without type II diabetes mellitus to identify factors holding prognostic values and implicated in mortality.

## Methods

This study protocol was approved by the Institutional Review Board of the Jiangxi Chest Hospital (no. 20190304). Informed consent was waived by the Institutional Review Board due to the retrospective nature of the study design.

### Study setting & participants

We retrospectively reviewed the medical records of patients with cryptococcal meningitis admitted to the Department of Internal Neurology, Jiangxi Chest Hospital. Between January 2012 and December 2018, HIV-negative cryptococcal meningitis patients with type II diabetes mellitus (*n* = 26, DM II group) and without type II diabetes mellitus (*n* = 52, non-DM II group) were enrolled. The inclusion criteria are including: confirmed cryptococcal meningitis patient with type II diabetes mellitus and without type II diabetes mellitus; Follow-up data were available. Patients receiving antifungal treatments prior to admission, those presenting recurrent cryptococcal meningitis, and aged< 18 years old were excluded from our study. The diagnosis of cryptococcal meningitis was based on positive results of cerebrospinal fluid culture, cerebrospinal fluid cryptococcal antigen test (IMMY, OK, USA), or positive cerebrospinal fluid India ink staining. Type II diabetes mellitus was defined according to the Chinese guidelines for diabetes mellitus: (1) random blood sugar test: a blood sugar level is 200 mg per deciliter (mg/dL) — 11.1 millimoles per liter (mmol/L)- or higher; (2) fasting blood sugar: a fasting blood sugar is 7 mmol/L or higher on two separate tests; (3) oral glucose tolerance test: a blood sugar level is more than 200 mg/dL (11.1 mmol/L) after 2 h [20].

### Data collection

For each patient, the following data were obtained: demographic information, sex, location, time of cryptococcal meningitis symptom emergence, time of cryptococcal meningitis confirmation, underlying diseases except type II diabetes mellitus, duration of type II diabetes mellitus course, antifungal regimen, clinical manifestations, laboratory findings, brain image, length of hospital stay (days) and outcome. Temporary drains, lumbar drains, or external ventricular drains for the control of increased intracranial pressure were also recorded. Brain image abnormalities were defined according to head computed tomography or magnetic resonance imaging scans as any parenchymal abnormality (including meningeal enhancement, bleeding, and strokes), and discharge data were used to determine the outcome. Two authors collected the clinical data, and these data were checked by the third author. Disagreements were resolved by discussion between two authors supervised by an expert.

### Statistical analysis

IBM SPSS (version 20, International Business Machines Corporation, New York, NY, USA) was used for statistical analysis, and the categorical data were reported as the means ± standard deviations or percentages. Comparisons between groups were performed by the chi-square test, Mann-Whitney U test and logistic regression. *P*-values less than 0.05 were considered statistically significant.

## Results

### Initial characteristics and laboratory findings

The demographic details, initial clinical symptoms and laboratory data of patients with and without type II diabetes mellitus are shown in Table 1. Hypertension was the most common in the type II diabetes mellitus group, while in the non-type II diabetes mellitus group, respiratory disease was most common. There was no significant difference in age and underlying diseases between the two groups. We also found no significant difference in the initial clinical symptoms between the type II diabetes mellitus and the non- type II diabetes mellitus group, except for visual disorders, which were more frequent in the latter group (28.54% vs. 11.54%, *p* = 0.038). As for the type of visual disorders, the most common between the two groups was blurred vision, accounting for 62.5% (*n* = 5) and 80% (*n* = 4) in the type II diabetes mellitus group and the non- type II diabetes mellitus group, respectively. Other visual impairment in type II diabetes group, including blindness (*n* = 1), the abduction of eye limited (*n =* 1) and diplopia(*n =* 1). Diplopia occurred in one patients in non-type II diabetes groups. The most common symptoms in the two groups were headache (96.15%), followed by fever (76.92%), nausea/vomiting (52.56%), conscious disturbance (21.79%), and visual disorders (15.38%).

**Table 1:** Univariate statistics and multivariate logistic regression analysis of patients’ characteristics and laboratory findings on admission

Characteristics	Univariate analysis			Multivariate analysis	
	DM II group (*n =* 26)	Non-DM II group (*n* = 52)	*P* value	(95% CI)	*P* value
Age (years)	57.31 ± 14.27	52.20 ± 15.84	0.173		
Sex: Male (n %)	12(46.15%)	29(55.77%)	0.423		
Rural area (n %)	21(80.77%)	44(84.61%)	0.667		
**Underlying conditions**					
Respiratory diseases (n %)	3(11.53%)	7(13.46%)	0.811		
Chronic kidney diseases (n %)	2(7.69%)	4(7.69%)	1.000		
Hepatitis B (n %)	3(11.53%)	3(5.77%)	0.367		
Hypertension (n %)	4(15.38%)	2(3.85%)	0.071	(0.026,1.109)	0.064
Autoimmune disease (n %)	3(11.53%)	4(7.69%)	0.575		
Hematology and oncology (n %)	0	1(1.92%)	0.477		
The duration before confirmed diagnosis (days)	27.00 ± 23.13	26.30 ± 18.93	0.501		
Duration of DM II course (years)	4.20 ± 3.34	–	–		
**Clinical parameters**					
Fever (n %)	21(80.77%)	39(75.00%)	0.569		
Headache (n %)	25(96.15%)	50(96.15%)	1.000		
Nausea/Vomiting (n %)	16(61.54%)	25(48.07%)	0.262		
Vision disorders (n %)	8(28.54%)	6(11.54%)	0.038*	(0.056,0.705)	0.012*
Auditory symptoms (n %)	2(7.69%)	4(7.69%)	1.000		
Seizure (n %)	3(11.54%)	3(5.77%)	0.367		
Conscious disturbance (n %)	8(30.77%)	9(17.31%)	0.175		
**CSF examinations**					
Opening pressure (mmH_2_O)	271.54 ± 100.78	234.23 ± 105.98	0.125		
CSF protein (mg/l)	1027.62 ± 594.16	705.72 ± 373.88	0.031*	(1.000,1.002)	0.016*
CSF glucose (mmol/l)	2.08 ± 1.01	2.30 ± 1.41	0.250		
Positive India ink stain (n %)	19(73.08%)	40(76.92%)	0.709		
Positive cryptococcal antigen (n %)	25(96.15%)	50(96.13%)	1.000		
Positive culture result (n %)	17(65.38%)	32(61.54%)	0.740		
**Brain images (CT or MRI)**					
Meningeal enhancement (n %)	10(38.46%)	22(42.30%)	0.745		
Hydrocephalus (n %)	2(7.69%)	4(7.69%)	1.000		
Cerebral infarction (n %)	4(15.38%)	6(11.54%)	0.632		
Abscess (n %)	2(7.69%)	2(3.84%)	0.468		
Normale^a^ (n %)	8(30.77%)	18(34.62%)	0.734		

*DM II* diabetes mellitus II, *CSF* cerebrospinal fluid, *CT* computed tomography, *MRI* magnetic resonance imaging, *CI* confidence interval

^a^Normal brain images on CT or MRI were defined as no obvious intracranial lesions demonstrated; *p < 0.05

With regard to the cerebrospinal fluid examinations, the type II diabetes mellitus group had a higher cerebrospinal fluid protein level (1027.62 ± 594.16 mg/l) than the non-type II diabetes group (705.72 ± 373.88 mg/l; [95% CI 1.000,1.002]; *p* = 0.016). There was no significant difference in opening pressure between the two groups (mean 271.54 vs. 234.23 mmH_2_O; *p* = 0.125). There was no significant difference in brain images between the two groups. Meningeal enhancement was the most common manifestation between two groups, which responsible for 38.46% in type II diabetes mellitus group and 42.30% in non-type II diabetes group, respectively. Among 78 patients, 49 patients were diagnosed based on culture, 39 patients were diagnosed based on culture cryptococcal antigen and positive India ink stain.

### Treatment and outcomes

All the patients were treated with systemic antifungal drugs, as shown in Table 2. The induction treatment included amphotericin B with flucytosine, lipid amphotericin B infusion with flucytosine, amphotericin B + fluconazole, and voriconazole ± flucytosine, there were no significant differences between the patients in the two groups who received each regimen. For the management of increased intracranial pressure, most patients received repeated lumbar puncture, and a temporary drain was administered in one type II diabetes mellitus patient and two without type II diabetes patients. For long-term cerebrospinal fluid drainage, permanent shunt devices were inserted in one type II diabetes mellitus patient and three non-type II diabetes group patients. The time of hospitalization in the type II diabetes mellitus group was longer than that in the non- type II diabetes mellitus group (59.58 vs. 42.88 days, *p* = 0.132, respectively). The death rate was higher in the type II diabetes mellitus group (6/26; 23.08%) than in the non-type II diabetes mellitus group (4/52; 7.69%, *p* = 0.055).

**Table 2:** Treatment and clinical outcomes of the two groups

Variables	DM II group (*N =* 26)	Non-DM II group (*N =* 52)	*P* value
**Treatment**			
AMB + 5FC (n %)	18 (69.23%)	41 (78.85%)	0.351
Lip AMB + 5FC (n %)	5 (19.23%)	6 (11.54%)	0.284
AMB + FCZ (n %)	0	3 (5.77%)	0.212
VCZ ± 5FC (n %)	3 (11.54%)	2 (3.84%)	0.191
Treatment for IICP (n %)	24 (92.30%)	47 (90.38%)	0.779
Repeated LP (n %)	20 (76.92%)	39 (75.00%)	0.852
Temporary drain (n %)	2 (7.69%)	5 (9.62%)	0.779
Permanent shunt device (n %)	1 (3.85%)	2 (3.85%)	1.000
Length of hospitalization (days)	59.58 ± 29.64	42.88 ± 1791	0.132
In-hospital mortality	6 (23.08%)	4 (7.69%)	0.055

Abbreviations: *DM II* type II diabetes mellitus, *AMB* amphotericin B, *5FC f*lucytosine, *Lip AMB* lipid amphotericin B, *FCZ* fluconazole, *VCZ* voriconazole, *IICP* increased intracranial pressure, *LP* lumbar puncture

We also analyzed possible factors associated with mortality between the surviving and dead cryptococcal meningitis patients with type II diabetes mellitus (Table 3). We found that at the initial presentation, nausea and vomiting was associated with mortality among type II diabetes mellitus patients (*p* = 0.027). Although poor type II diabetes mellitus management was not associated with mortality, 66% of patients who died did not manage diabetes well, compared with 30% in surviving patients.

**Table 3:** Possible risk factors associated with mortality among CM patients with DM II

Variables	Surviving patients (*N* = 20)	Dead patients (*N* = 6)	*P* value
Age (years)	57.95 ± 15.38	55.75 ± 10.61	0.533
Sex: Male (n %)	9 (45.00%)	3 (50.00%)	0.829
Duration before confirmed diagnosis (days)	29.85 ± 22.80	17.83 ± 11.05	0.355
Duration of DM II course (years)	4.27 ± 3.67	4.00 ± 2.19	0.882
Poor DM II management (n %)	6 (30.00%)	4 (66.67%)	0.105
Underlying diseases (n %)	11 (55.00%)	4 (66.67%)	0.612
**Clinical parameters**			
Fever (n %)	15 (75.00%)	6 (100%)	0.671
Headache (n %)	19 (73.08%)	6 (100%)	0.419
Nausea/Vomiting (n %)	10 (50.00%)	6 (100%)	0.027*
Vision disorders (n %)	3 (15.00%)	1 (16.67%)	0.921
Auditory symptoms (n %)	3 (15.00%)	1 (16.67%)	0.921
Seizure (n %)	2 (10.00%)	1 (16.67%)	0.654
Conscious disturbance (n %)	5 (25.00%)	3 (50.00%)	0.245
**CSF examinations**			
Opening pressure (mmH_2_O)	258.00 ± 99.92	316.67 ± 98.20	0.295
CSF protein (mg/l)	983.35 ± 641.03	1175.20 ± 411.96	0.268
CSF glucose (mmol/l)	2.38 ± 1.13	1.95 ± 0.40	0.882
Brain images abnormality^a^ (n %)	13 (65.00%)	5 (83.33%)	0.393

Abbreviations: *CM* cryptococcal meningitis, *DM II* type II diabetes mellitus, *CSF* cerebrospinal fluid

^a^Brain image abnormalities included meningeal enhancement, hydrocephalus, cerebral infarction and so on

*p < 0.05

## Discussion

Cryptococcal meningitis is a common opportunistic central nervous system infection that can occur in both immunocompromised and immunocompetent patients [21]. Many underlying diseases and conditions, such as HIV infection, organ transplantation, autoimmune disease, chronic kidney disease, and long-term steroid use, are reported as risk factors for *Cryptococcus* infection [4, 5, 11, 22]. Thus, understanding the clinical characteristics of cryptococcal meningitis patients suffering from secondary complications is important for clinical management and reducing mortality.

In this study, we found that cryptococcal meningitis patients with type II diabetes mellitus were older and more often presented with visual disorders when compared to cryptococcal meningitis patients without type II diabetes mellitus. In cerebrospinal fluid examinations, more patients had higher opening pressure and cerebrospinal fluid protein levels. In addition, the time of hospitalization was longer and mortality was higher in the type II diabetes mellitus group than in the non-type II diabetes mellitus group.

In both groups, the most common symptoms of the 78 patients were fever, headache, nausea, vomiting and consciousness disturbance, which were partially consistent with data reported in previous studies [3]. There were no significant differences in the above mentioned symptoms between both of the groups, with the exception of vision disorders. The reasons for vision disorders include: diabetes itself can cause visual impairment, some patients have visual impairment in the diabetic stage but worsen after cryptococcal meningitis, and the other is simply caused by cryptococcal meningitis. Considering that diabetes itself can cause visual disturbance, perhaps this is the main reason for the difference between two groups [23]. In addition, we found that type II diabetes mellitus patients presented with higher intracranial pressure. Although higher intracranial pressure has been associated with mortality among cryptococcal meningitis patients [24, 25], we did not observe a direct relationship between intracranial pressure and mortality in the present study, probably because a ventriculoperitoneal shunt or temporary drain was used in patients with intracranial pressure or because mannitol is used as medication to reduce intracranial pressure.

Among the included 78 cryptococcal meningitis cases, 75% patients (*n* = 59) received standard antifungal therapy. The antifungal regimen in the two groups was not significantly different. Five patients in the two groups were treated with voriconazole combined with flucytosine or voriconazole alone and achieved good results. Voriconazole has been found to be an effective drug for cryptococcal meningitis in some studies [26, 27]. Although requires larger patient population, our study showed that voriconazole in combination with flucytosine might be a useful alternative therapy for cryptococcal meningitis patients.

Cryptococcal meningitis patients with type II diabetes mellitus showed higher mortality compared with non-type II diabetes mellitus group. In the type II diabetes mellitus group, 73.07% of patients were > 60 years old, and as the prevalence of diabetes mellitus also increases in the aged population, age-associated reductions in functional immune status may be a risk factor for mortality [28]. As shown in Table 3, the presentation of nausea and vomiting was a possible risk factor for mortality type II diabetes mellitus patients. The presence of nausea and vomiting may indicates patients with higher opening pressure, which has been reported to be associated with poor prognosis [24]. We found that 50% of type II diabetes mellitus patients had a more than 5-year history of type II diabetes mellitus, and 38% of type II diabetes mellitus patients (*n* = 10) were reported to be untreated or had poor control of blood glucose despite treatment. We also found that more than half of the patients who died had a history of uncontrolled or poorly controlled blood glucose levels, indicating that blood glucose management may be a probable cause of the poor prognosis of cryptococcal meningitis, as shown in previous studies [29–31].

There are several limitations of this study. First, this was a retrospective study performed in a single hospital by reviewing medical records. Secondly, we could not appropriately assess the impact of voriconazole therapy on mortality, which requires dedicated studies with large patient numbers. Thirdly, according to the different serotypes of *Cryptococcus*, its manifestations and prognosis are different, but this was not further assessed in this study. Further large prospective multicenter studies are needed to improve the generalizability of the data on cryptococcal meningitis .

## Conclusion

In conclusion, our study demonstrated that cryptococcal meningitis patients with type II diabetes mellitus differ significantly from patients without type II diabetes mellitus with respect to clinical symptoms such as visual disorders and cerebrospinal fluid protein levels. In addition, it is necessary to note that type II diabetes mellitus patients experienced higher opening pressure, higher glucose and longer hospitalization than non- type II diabetes patients. Our data also suggested that nausea and vomiting could be related to mortality in patients with type II diabetes mellitus. We need to highlight the importance of type II diabetes mellitus management, considering that the majority of cryptococcal meningitis patients with type II diabetes mellitus who died had poorly controlled blood glucose levels.

## Data Availability

The datasets used and analyzed during the current study are available from the corresponding author on reasonable request.

## Abbreviations

CM: Cryptococcal meningitis
DM II: Type II diabetes mellitus
CI: Confidence interval
CSF: Cerebrospinal fluid
IICP: Increased intracranial pressure
CT: Computed tomography
MRI: Magnetic resonance imaging
AMB: Amphotericin B
5-FC: Flucytosine
lip AMB: Lipid amphotericin B infusion
FCZ: Fluconazole
VCZ: Voriconazole
